# Structural Analysis of PTM Hotspots (SAPH-ire) – A Quantitative Informatics Method Enabling the Discovery of Novel Regulatory Elements in Protein Families* [Fn FN2]

**DOI:** 10.1074/mcp.M115.051177

**Published:** 2015-06-12

**Authors:** Henry M. Dewhurst, Shilpa Choudhury, Matthew P. Torres

**Affiliations:** From the ‡Georgia Institute of Technology; School of Biology; 310 Ferst Drive; Atlanta, Georgia 30332

## Abstract

Predicting the biological function potential of post-translational modifications (PTMs) is becoming increasingly important in light of the exponential increase in available PTM data from high-throughput proteomics. We developed *s*tructural *a*nalysis of *P*TM *h*otspots (SAPH-ire)—a quantitative PTM ranking method that integrates experimental PTM observations, sequence conservation, protein structure, and interaction data to allow rank order comparisons within or between protein families. Here, we applied SAPH-ire to the study of PTMs in diverse G protein families, a conserved and ubiquitous class of proteins essential for maintenance of intracellular structure (tubulins) and signal transduction (large and small Ras-like G proteins). A total of 1728 experimentally verified PTMs from eight unique G protein families were clustered into 451 unique hotspots, 51 of which have a known and cited biological function or response. Using customized software, the hotspots were analyzed in the context of 598 unique protein structures. By comparing distributions of hotspots with known *versus* unknown function, we show that SAPH-ire analysis is predictive for PTM biological function. Notably, SAPH-ire revealed high-ranking hotspots for which a functional impact has not yet been determined, including phosphorylation hotspots in the N-terminal tails of G protein gamma subunits—conserved protein structures never before reported as regulators of G protein coupled receptor signaling. To validate this prediction we used the yeast model system for G protein coupled receptor signaling, revealing that gamma subunit–N-terminal tail phosphorylation is activated in response to G protein coupled receptor stimulation and regulates protein stability *in vivo*. These results demonstrate the utility of integrating protein structural and sequence features into PTM prioritization schemes that can improve the analysis and functional power of modification-specific proteomics data.

Post-translational modifications (PTMs)^1^ are a rapidly expanding and important class of protein feature that broaden the functional diversity of proteins in a proteome. By definition, PTMs change protein structure and therefore have the potential to affect protein function by altering protein interactions, protein stability or catalytic activity (1, 2). As they have been found to occur on nearly every protein in the eukaryotic proteome, PTMs broadly impact nearly all known cellular processes. Over 300 different types of PTM are known, ranging from single atom modifications (*e.g.* oxide) to small protein modifiers (*e.g.* ubiquitin), which can occur on all but five amino acid residues resulting from enzymatic or nonenzymatic processes (3). Over 220,000 distinct PTM sites have been experimentally identified across ∼77,000 different proteins to date (dbPTM; http://dbptm.mbc.nctu.edu.tw/statistics.php) – numbers that continue to grow exponentially because of improved methods for high throughput detection by mass spectrometry (MS). By virtue of how they are detected, most PTM data are sequence-linked and lack structural context.

The function of most PTMs is unknown because the rate of PTM detection far surpasses the rate at which any one modification can be studied empirically. Moreover, the functional impact of every PTM is likely not equivalent (4). For example, computational analysis of phosphorylation sites in yeast and human proteomes indicate that well-conserved phosphosites are more likely to have a functional consequence compared with poorly conserved sites, yet only a fraction of phosphosites are well conserved (5, 6). Consequently, the development of tools that provide functional prioritization of PTMs could have a broad impact on our understanding of protein regulation, biological mechanism, and molecular evolution.

The emerging need for methods that predict the functional impact of a PTM has not yet been met. Longstanding methods capitalize predominantly on the sequence context of PTMs and have been used to predict sites of modification (expasy.org/proteomics/post-translational_modification) and to compare enzyme/substrate interactions (7–9). More recently, studies aimed at expanding the parameters associated with functional PTMs have emerged. In these cases, a set of common features correlated with functional importance are derived from the analysis of PTMs within and between organisms including: number of PTM observations at a multiple sequence alignment position (*i.e.* hotspots), measures of co-occurrence between different PTMs (*e.g.* distance between phosphorylation and ubiquitination sites), biological dynamics (up or down-regulation), and protein–protein interaction influence (7, 10–12). Recent efforts to provide structural context by linking individual PTMs to three-dimensional structures in the protein data bank (PDB) have also been described (13, 14). However, these resources are extensions of existing PTM databases that allow visualization of single instances of modification onto individual proteins, but do not provide quantitative or analytical value.

In principle, combining PTM hotspot and structural analysis would offer multiple advantages over any one approach used in isolation. Sequence homology provides protein family membership—thereby clustering PTMs into hotspots for groups of proteins to provide information about: (1) the evolutionary conservation and (2) observation frequencies of PTMs within the family. A primary consequence of their sequence homology is that members of a protein family will exhibit similar structures and protein interactions—features that dictate the function of protein systems. A secondary consequence is that PTM hotspots generated by alignment can be projected onto family-representative protein structures, which places each PTM hotspot into a three-dimensional context that can be visualized for each family. The structural context enabled by this projection can also provide spatial information about the PTM site that can supplement the sequence characteristics of the hotspot, namely: (3) solvent accessibility, which provides an estimate of whether a modification could occur on the folded protein; and (4) protein interface residence, which indicates the potential of the PTM to disrupt protein–protein interactions. Despite the theoretical advantages, no single tool has been developed that exploits the quantitative output from both sequence and structural data to evaluate the function potential of PTMs.

Here we describe a new analytical method – *S*tructural *A*nalysis of *P*TM *H*otspots (SAPH-ire), which ranks PTM hotspots by their potential to impact biological function for distinct protein families (Fig. 1). We demonstrate the application of SAPH-ire to the complete set of PTMs for eight distinct protein families including large heterotrimeric G proteins—revealing high-ranking hotspots for which a biological function has not yet been determined. In particular, SAPH-ire revealed the N-terminal tail (Nt) of G protein gamma (Gγ) subunits as one of the highest ranking PTM hotspots for heterotrimeric G proteins (Gα, Gβ, and Gγ). We tested this prediction by monitoring the phosphorylation state and mutation effects of phosphorylation sites in the N terminus of the yeast Gγ subunit (Ste18). Consistent with SAPH-ire predictions, we found that phosphorylation of Ste18-Nt is biologically responsive to a GPCR stimulus and that phospho-null or phospho-mimic mutation of these sites controls protein abundance in an opposite manner *in vivo*. Thus, SAPH-ire is a powerful new method for predicting the function potential of PTM hotspots, which can guide empirical research toward the discovery of new protein regulatory elements based on high-throughput proteomics.

**Fig. 1.**
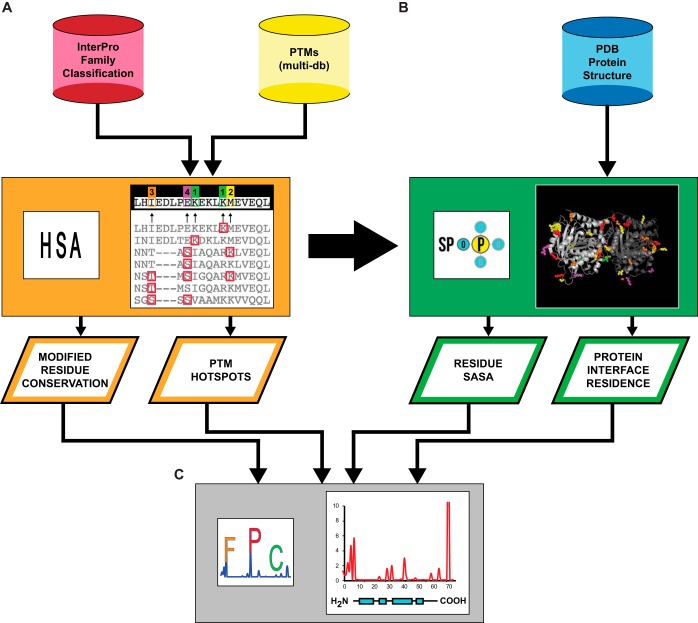
**Schematic diagram of the SAPH-ire method.** *A*, SAPH-ire integrates InterPro, the Protein Data bank (PDB) and a customized database of experimentally validated PTMs. Uniprot entries with PTMs that belong to specific InterPro-classified protein families undergo multiple-sequence alignment (MSA) and PTM hotspot analysis (HSA), which layers all PTMs for a given alignment position in the MSA. The total PTMs observed in each hotspot and the conservation of a modifiable residue (*e.g.* conservation lysine at a ubiquitination hotspot) at the hotspot are quantified. *B*, PTM hotspots within the protein family are then projected onto all known crystal structures for the family using the Structural Projection of PTMs (SPoP) tool. From the structural topology of PTM hotspots generated by SPoP, the solvent accessible surface area (SASA) and protein interface residence is quantified for each hotspot. *C*, PTM Function Potential Calculator (FPC) integrates the output from HSA and SPoP, resulting in PTM function potential scores for each hotspot. The function potential score can be used to rank PTM hotspots within or between protein families – prioritizing hotspots with the greatest potential to be biologically regulated and/or effect a biological function for the protein family of interest.

## EXPERIMENTAL PROCEDURES

### 

#### 

##### SAPH-ire

SAPH-ire is comprised of three modular components: one, multiple sequence alignment (MSA) and PTM hotspot generation; two, structural projection of PTM hotspots; and three, hotspot ranking via the function potential (FP) calculation. Multiple sequence alignment is achieved using the MUSCLE algorithm with default parameters (15). Automated structural projection of PTM hotspots and FP scoring are achieved using two customized programs built in our lab (Structural Projection of PTMs or SPoP, and FP-calculator or FPC). PTMs used in SAPH-ire are first compiled into a local MySQL database of PTMs from 12 independent sources (see supplemental Table S1), in which only entries from eukaryotic organisms are selected for analysis and predicted modification sites are excluded from the gathered data. Protein family membership was determined using InterPro classification (http://www.ebi.ac.uk/interpro/).

Protein family members are organized in three steps. First, all members with experimentally observed PTMs or a structure deposited in the RSCB PDB undergo multiple sequence alignment (MSA) and PTMs independently identified on specific residues within each member of the family are layered with respect to their positions in the alignment – creating PTM hotspots (Fig. 1). Second, a target sequence within the alignment is chosen based on the availability of an associated protein structure in the PDB. Example protein family/uniprot ID/PDB structures used for demonstration here are: Gα/P10824/1GP2; Gβ/P62871/1GP2; Gγ/P63212/1GP2; α-tubulin/P09733/4FFB; β-tubulin/P02557/4FFB; Ras/P01112/1BKD; Rho/P61586/1TX4; Rab/P62491/1OIV (supplemental File S3). Using Structural Projection of PTMs (SPoP), the PTM hotspots can be projected onto any available protein structure in the alignment. For visualization purposes, PTM projections are colored based on the frequency of PTM observation – where the default model color represents no observed PTMs, green for one, yellow for two, orange for three, magenta for four, red for five or more. Third, using FP-calculator (FPC), FP scores are calculated for each PTM hotspot within the family, which combines the total observed PTMs at an alignment position, the weighted solvent accessibility of the residue within the projected structural target, the protein interface residence, and sequence conservation within the MSA. Solvent accessibility is calculated with the POPS algorithm (16). To ensure that protein interfaces were not impacting solvent accessibility values, the POPS algorithm was run on isolated, single chains from each structure. For residues not resolved by crystallography (*e.g.* terminal tails), the maximum solvent accessible surface area was assigned if the structural element was determined to be disordered by DisEMBL (17). Interface residence was determined using a custom-filtered version of ProtInDB (http://protindb.cs.iastate.edu/index.py) that excludes chimeric protein structures and interfaces contributed by artificial same-chain interactions resulting from crystallography.

The goal of the SAPH-ire method is to integrate PTM, protein sequence and protein structure data to calculate FP scores for each PTM hotspot in a protein family. The FP score is based on weighted criteria designed to diminish the rank of PTMs not expected to be biologically reasonable for a protein in its folded state as determined through crystallographic or other form of hi-resolution three-dimensional structural evidence (*e.g.* NMR) available in the PDB. First, higher FP is expected for PTMs that are more frequently observed (*i.e.* total PTMs per projected residue; aka: hotspot intensity). Second, high FP is expected at residues with higher solvent accessible surface area (*SASA*) in the folded protein structure. This criteria considers that biologically relevant PTMs are more frequently observed as a result of enzymatic catalysis that requires accessibility of the modified residue on a properly folded protein. Although PTMs found buried deep within a folded protein structure might have functional value, they are less likely to be involved in regulating a properly folded and functional protein. Third, higher FP is expected for PTMs that reside at protein interfaces or at highly accessible surfaces that have potential to participate in protein–protein interactions. PTMs found at protein interfaces have the potential to change protein interactions and therefore biological function, whereas highly exposed PTM sites may represent protein interfaces yet to be identified. Fourth, high FP is expected for PTMs on well-conserved amino acid residues because they are more likely to be critical for protein structure and function. FP score is calculated as follows:

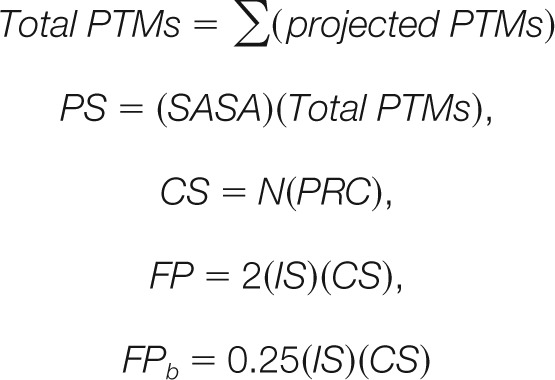
 where *PS* refers to *PTM Score*; *SASA* is the structurally derived (as opposed to sequence-derived) solvent accessible surface area; IS (interface score) is the PTM score weighted if the hotspot residue is found at a protein interface; *CS* is the weighted residue conservation score; *PRC* (Ptm_Res_Con) is the percent conservation of a modifiable residue at the projected PTM site (*e.g.* percentage of serine threonine or tyrosine conserved at projected sites of phosphorylation, lysine for ubiquitination, etc.), and *FP* is the function potential score for residues at protein interfaces and exposed surfaces (FP) or at solvent inaccessible surfaces (*FP_b_*) as determined from the SASA output from the POPS algorithm applied to the isolated target protein structure (*i.e.* in the absence of other proteins).

##### Yeast Strains and Plasmids

Standard methods for growth, maintenance, and transformation of yeast were used throughout. Strains used or created for this study are shown (see supplemental Table S5). Yeast strain *BY4741* (*MATa leu2*Δ *met15*Δ *his3*Δ *ura3*Δ) was used as the isogenic wild-type strain in all experiments. Genomic modification (point mutation or epitope tagging) of BY4741 was accomplished by PCR-mediated disruption using the dellito perfetto method (18). Ste18 harbors three phosphorylation-acceptor residues in the N-terminal tail: threonine-2, serine-3, and serine-7. Two of the three residues (serine 7 and either threonine-2 or serine-3) have been observed in the phosphorylated state by mass spectrometry (19). To introduce phospho-null (3A; T2A/S3A/S7A) or phospho-mimic (3E; T3E/S3E/S7E) mutations into Ste18, the entire open reading frame of *STE18* was precisely replaced with the CORE cassette amplified from the pCORE plasmid with chimeric oligonucleotides (see supplemental Table S4), containing flanking sequences to the *STE18* ORF. Point mutations were introduced into STE18 contained in plasmid pZM552-STE18 (www.dnasu.org) using a QuikChange mutagenesis kit (Life Technologies, Carlsbad, CA) followed by PCR amplification, transformation and CORE popout selection on 5-FOA plates. An epitope tag (Hemagglutinin; HA) was inserted after the initiator methionine of STE18 using HA-encoded oligonucleotides using the dellito perfetto method, wherein CORE was introduced at the N terminus of the STE18 gene and later replaced with a single HA tag (see supplemental Table S4). Strain construction was verified by PCR amplification and dideoxy sequencing (Eurofins MWG Operon).

##### Yeast Cell Culture and Immunoblotting

All experiments were conducted using YPD growth medium at 30C with shaking at 250 rpm and cell culture density was determined by absorbance at 600 nm. Single colonies from freshly plated yeast strains were inoculated into 3 ml starter cultures and grown to saturation overnight. The next morning, cultures were diluted to OD 0.1 in 10 ml and grown to OD 0.6–0.8, then diluted to OD 0.001 in 75 ml and grown overnight to OD 0.6–0.8 followed by treatment with α-factor peptide pheromone (Genscript) at 3 μm final concentration. For each time point, 10 ml aliquots were transferred from the culture vessel to conical tubes containing 0.5 ml trichloroacetic acid (TCA) on ice. Cells were harvested by centrifugation at 3724 × *g* Allegra X-14R Beckman Coulter Centrifuge, washed with 1 ml Milli-Q water and transferred to microfuge tubes that were immediately frozen at −80°C.

Proteins were extracted using TCA and suspended in neutralizing SDS re-suspension buffer as previously described (20). The concentration of protein extracts was determined using DC protein assay reagent (BioRad, Hercules, CA) followed by normalization in SDS-PAGE loading buffer. Samples were analyzed by 12.5% SDS-PAGE and transferred to nitrocellulose membranes. All immunoblots were conducted in 1x tris-buffered saline with 1% Tween-20 (TBS-T) with 5% nonfat dry milk. Immunoblot analyses of HA tagged Ste18 or G6PDH loading control were accomplished using mouse monoclonal anti-HA antibodies (Sigma, Cat#H3663; 1:2000, 2 h) and rabbit polyclonal anti-G6PDH (Sigma Cat#A9521; 1:50,000, 1 h). HRP-conjugated secondary antibodies (goat-anti-rabbit or anti-mouse) were used for chemiluminescent detection using ECL reagent (Perkin Elmer Cat # NEL 104001EA). Immunoblots were quantified by high-resolution scanning and pixel densitometry using ImageJ software as described previously (21).

##### Statistical Analysis

Statistical analysis of SAPH-ire data output as well as for quantified MAPK and HA immunoblots was achieved using GraphPad Prism software version 6.

## RESULTS

### 

#### 

##### Combining Sequence and Structural Characteristics of Modified Residues Distinguishes PTM Hotspots with a Known Biological Function

SAPH-ire was originally developed for the analysis of PTMs on large heterotrimeric G proteins because of the abundance of structural, mutational, functional and PTM data as well as their medical significance. Large heterotrimeric G proteins form a very well characterized protein complex represented by three different protein families (Gα, Gβ, and Gγ). The complex is activated by G protein coupled receptors (GPCRs), which can be stimulated by a wide-array of extracellular ligands including hormones, neurotransmitters, pheromones, light, among other compounds (22, 23). Activated Gα subunits undergo a conformational change coupled with the exchange of bound guanine nucleotide diphosphate (GDP) for the triphosphate form (GTP). Activated, GTP-bound Gα no longer interacts with Gβγ heterodimers, which allow Gα and Gβγ to interact with effector proteins that promote intracellular signaling cascades essential for response to the GPCR stimulus (24). As the most widely exploited drug targets in the pharmaceutical industry, GPCRs and heterotrimeric G proteins play a major role in human development, neurology, and disease (25–27).

For comparative purposes, and to demonstrate that SAPH-ire can be used to study PTMs for any protein family, we analyzed PTMs for heterotrimeric G proteins and five additional G protein families. These included the small G proteins Ras, Rho, and Rab, as well as the structural G proteins α and β tubulin—for which there exists an abundance of structure, function, interaction and PTM data. First, we compiled a comprehensive MySQL database of nonredundant and experimentally verified PTM data for each G protein family across 12 different public PTM databases (see supplemental Table S1). A total of 1728 individual PTMs (characterized by specific modification type, protein target and residue location) were identified with these constraints and included in the analysis (see supplemental Table S2). For each family, we mined published reports to identify the subset of PTMs for which biological responsiveness (*i.e.* a change in modification state accompanied by a change in condition) and/or biological function (*i.e.* a change in phenotype accompanied by modification site mutation) had been shown experimentally (see supplemental Table S3). Multiple sequence alignment (MSA) of modified protein sequences produced a list of total PTMs found at each alignment position in the family—resulting in 451 PTM hotspots, 51 of which have been shown to exhibit biological responsiveness or function.

**Fig. 2.**
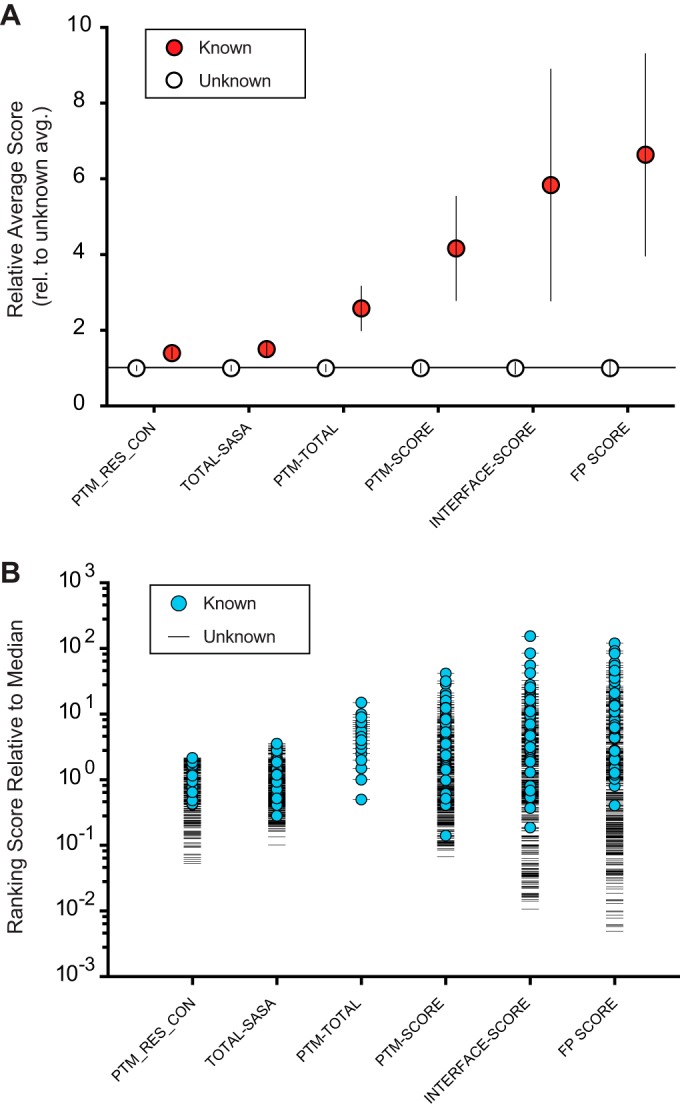
**Combining sequence and structural characteristics of modified residues distinguishes PTM hotspots with a known biological function.** The ranking of PTM hotspots for the eight G protein families was compared using different ranking factors (x-axes) and PTM hotspots with known function were then compared with those of unknown function. *A*, Average rank scores were normalized relative to the average score for PTM hotspots with unknown function (unknown). Bars represent the 95% confidence intervals of the mean. All points on the plot were found to be significantly different from one another using a Mann-Whitney nonparametric significance test. *B*, Individual data points used in panel *A* show that function potential scoring has a pronounced effect on the distribution of rank scores from the hotspots with unknown function, but less of an effect on the distribution of rank scores for hotspots with a known function. Each column of data in panels *A* and *B* are generated from the same hotspot data. Detailed statistics can be found in Supplemental File S1.

Next, we ranked each PTM hotspot with respect to sequence character (modifiable residue conservation—“ptm_res_con”), structural character (total solvent accessible surface area for the hotspot residue—“total sasa”), or PTM hotspot intensity at each alignment position (“total PTMs”). To determine the effectiveness of each ranking method, we compared the PTM hotspots with known function (“Known”) to those with unknown function (“Unknown”), working under the assumption that an effective ranking method should provide maximal separation between the two categories. Ranking hotspots by modifiable residue conservation provided a minor, yet statistically significant difference between hotspots with known *versus* unknown function (Fig. 2*A* and supplemental File S1). These results are consistent with previous reports that the conservation of sites with known function is more constrained than for those with unknown function (6). Ranking hotspots by the total solvent accessible surface area of the modified residue also provided a small (1.5-fold) but statistically significant difference between “known” *versus* “unknown” hotspots. Comparatively, ranking by total PTMs found at an alignment position (*i.e.* hotspot analysis) provided a 2.4-fold difference between the mean for known *versus* unknown hotspots.

Although ranking hotspots by individual parameters (*e.g.* PTM residue conservation) was somewhat effective, the distinction between “known” *versus* “unknown” hotspots was small if not indistinguishable. In contrast, ranking PTM hotspots by any combination of sequence and structural character resulted in a statistically significant four to sixfold difference in known *versus* unknown ranks (Fig. 2*A* and supplemental File S1). In particular, FP scoring, which combines total PTMs and accessible surface area with protein interface residence and modifiable residue conservation, was the most effective of all the ranking methods. In comparison to other combinatorial ranking methods, FP scoring specifically improved the separation between the rank score distributions for known and unknown PTM hotspots (Fig. 2*B*). Thus, by integrating both sequence and structural data, FP scoring by SAPH-ire improves the prioritization ranking of PTM hotspots compared with hotspot analysis alone.

**Fig. 3.**
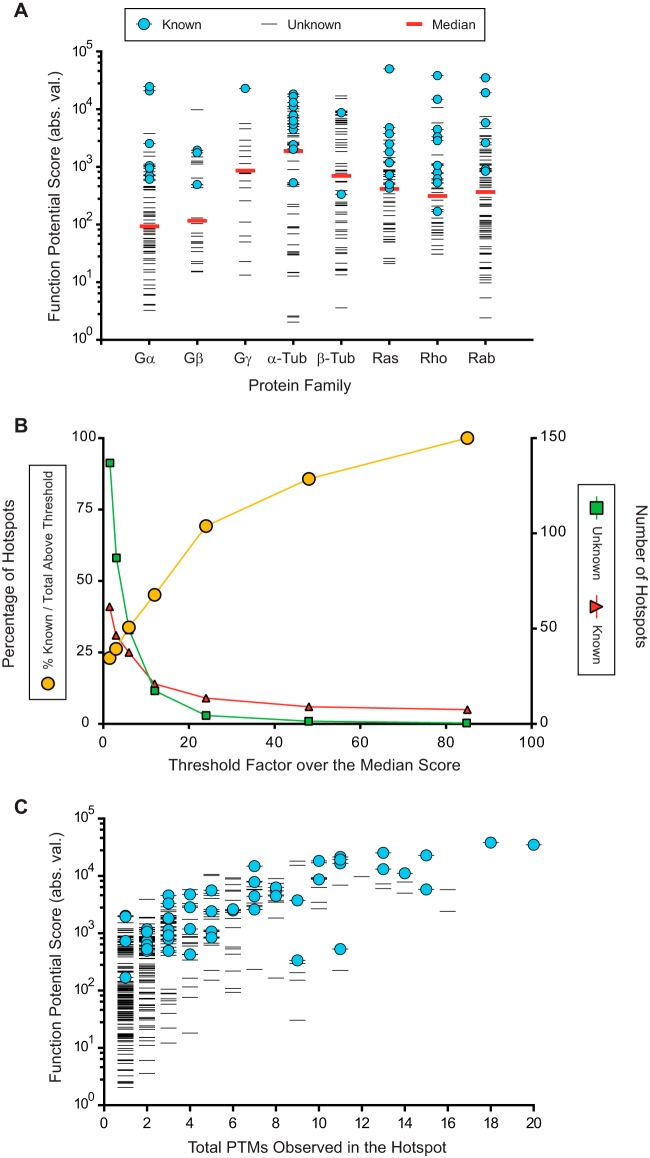
**Function potential scoring by SAPH-ire is more effective than total PTM analysis for distinguishing PTM hotspots with known function.** Function potential scores for PTM hotspots with known or unknown function were compared between the eight G protein families. *A*, Rank ordered FP scores for PTM hotspots with known and unknown function are shown. The median score for each family is shown (red line). Ninety-Four percent of hotspots with a known function were found to segregate above the median FP score. *B*, Quantitative comparison of hotspots with known *versus* unknown function. The number of “known” and “unknown” hotspots is shown at a given threshold over the family median score (right *y* axis). The percentage of PTM hotspots with known function relative to total hotspots (known/[known+unknown]) increases as the FP score increases (left *y* axis). *C*, Correlation plot between function potential score (*y* axis) and hotspot intensity (*x* axis) shows hotspots with known function are clustered by function potential scoring, but not by hotspot intensity.

Comparisons between heterotrimeric G proteins, tubulins, and the small G proteins Ras, Rho, and Rab suggest that FP scoring is family independent. PTM hotspots with a known biological function exhibited FP scores higher than the median value in greater than 94% of the 51 cases studied here (Fig. 3*A*). Moreover, we found that the percentage of PTM hotspots with a known function increases proportionally with FP score (Fig. 3*B*). In some hotspots, PTMs with a known function have been observed frequently (*i.e.* exhibit high total PTM value at the hotspot), which is reflected in FP scoring. However, we found many exceptions to this trend where PTM hotspots with a known function contained as few as one observation of the PTM (Fig. 3*C*). Thus, FP scoring by SAPH-ire is effective at ranking PTM hotspots in a manner that is independent of protein family.

##### SAPH-ire Predicts High Function Potential for Phosphorylation Hotspots in the N-terminal Tails of Gγ Subunits

**Fig. 4.**
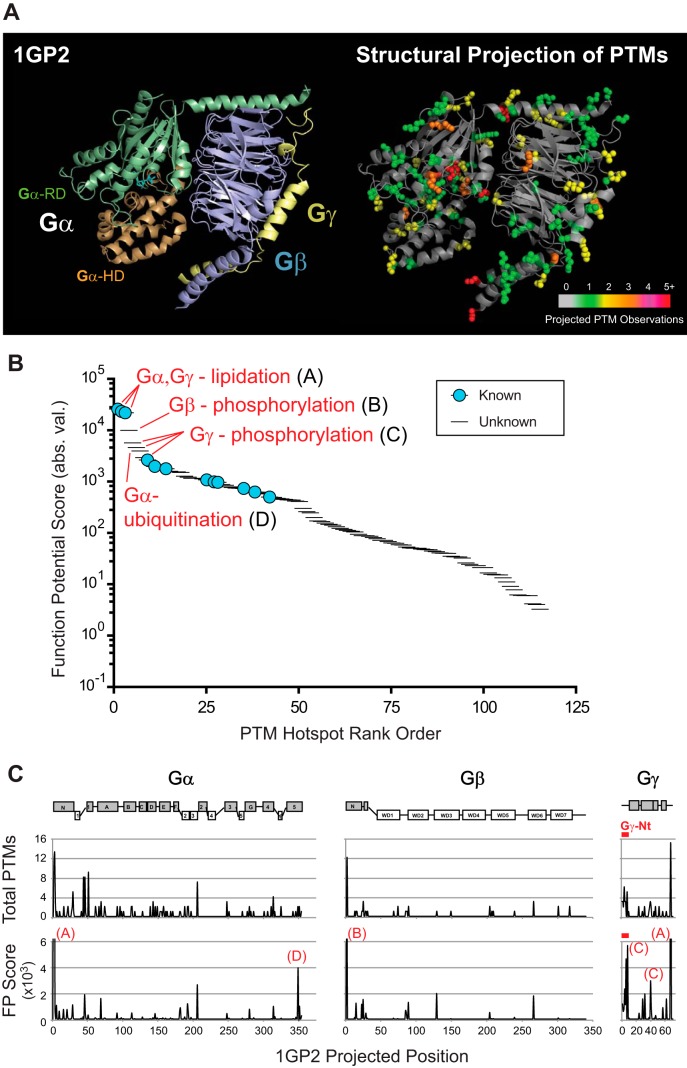
**SAPH-ire predicts high function potential hotspots in the N-terminal tails of Gγ subunits.** *A*, Structural projection of PTM hotspots onto the crystal structure of a canonical heterotrimeric G protein. Total PTMs observed in each hotspot are indicated by color code. For visualization purposes, sidechains with projected hotspots are shown with van der Waals radii (vdw = 1.0). *B*, Rank ordered comparison of FP scores for Gα, Gβ, and Gγ combined. The top eight hotspots are labeled. *C*, Total PTMs (above) or FP score (below) plotted with respect to the primary structure of Gα, Gβ and Gγ subunits. Hotspots labeled in panel *B* are shown. Red dash indicates the position of Gγ-Nt. Crystal structure 1GP2 was used as the projection target in all panels. (RD) Ras-like domain. (HD) Helical domain.

Using the SAPH-ire method, comprehensive and family-wide PTM data can be visualized in the context of a representative protein structure. In the case of heterotrimeric G protein families, we utilized the 1GP2 crystal structure consisting of a heterotrimeric complex between Gαi1 (*Rattus norvegicus*, P10824), Gβ1 (*Bos taurus*, P62871), and Gγ2 (*Bos taurus*, P63212) (Fig. 4*A*, left) (28). To facilitate visualization, PTM hotspot sidechains are shown with atomic van der Waals spheres and color-coded based on the total number of PTMs observed at each hotspot (Fig. 4*A*, right). A total of 115 PTM hotspots corresponding to 281 individual PTMs were identified by SAPH-ire across the three families (*see* supplemental Table S2). In order to determine the highest-ranking PTM hotspots in the heterotrimeric complex we rank ordered FP scores for Gα, Gβ, and Gγ protein families together. We found only ten out of 115 hotspots (8.7%) have been shown to be biologically responsive and/or functional experimentally (Fig. 4*B*). The highest-ranking hotspots correspond to palmitoylation and myristoylation sites localized to the extreme N termini of Gα subunits as well as a prenylation/palmitoylation hotspot found in the C termini of all Gγ subunits (Fig. 4*B*, (A)). Each lipid modification is essential for anchoring the heterotrimer to the plasma membrane and each is therefore critical for signal transduction (see supplemental Table S3). The next five hotspots (fourth through eighth) have no previously assigned function and correspond to an N-terminal phosphorylation hotspot in Gβ subunits (B), three phosphorylation hotspots in Gγ subunits (C), and a ubiquitination hotspot in the C terminus of Gα subunits (D). To visualize the distribution of hotspots along the sequence length of each family, we plotted total PTMs or FP score along the primary structure of each subunit, revealing multiple high-FP hotspots localized to the short N-terminal tails of Gγ subunits (Gγ-Nt) (Fig. 4*C*).

**Fig. 5.**
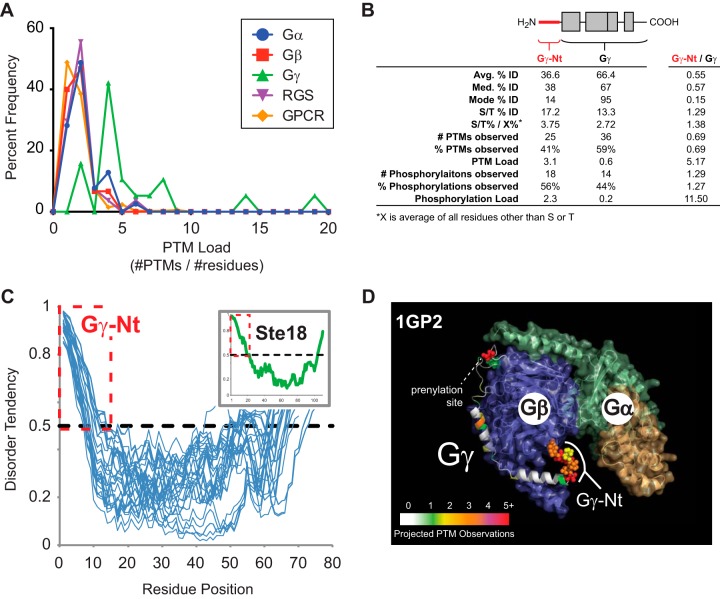
**Intrinsic structural disorder and phosphorylation are conserved in Gγ N-terminal tails.** *A*, Frequency distribution of PTM load for six different protein families essential for G protein signaling – Gα, Gβ, Gγ, RGS protein, and GPCR. PTM load calculated by dividing total number of PTMs observed for a uniprot entry by the number of residues in the full-length protein. *B*, Table of protein sequence identity, PTM load and phosphorylation load within the Gγ family. For comparison, sequence identity and PTM statistics for Gγ-Nt have been compared with the remainder of the Gγ protein (Gγ-Nt/Gγ). *C*, Disorder tendency for all reviewed Uniprot entries for Gγ subunits (blue lines). Yeast Gγ/Ste18 (inlay). Structural disorder is predicted for tendency values greater than 0.5 using IUPred (29). *D*, 3D structure of mammalian heterotrimeric G protein (1GP2) with a threaded model of Gγ-Nt and PTM heatmap. Sidechains with projected hotspots are shown with van der Waals radii (vdw = 1.0).

To our surprise, we found that the Gγ family exhibits twice the PTM load (#PTMs/protein length) of any other heterotrimeric G protein family in addition to GPCRs and RGS protein families (Fig. 5*A*). Unexpectedly, 41% of all Gγ PTMs are located within Gγ-Nt – 18 of which correspond to 56% of all observed phosphorylations in the Gγ family (Fig. 5*B*). In addition, we found that 100% of the N-terminal tails from Gγ family members contain multiple serine and/or threonine residues, which are enriched by 1.4 times the next most abundant residue. The N-terminal tails of Gγ subunits have not been resolved by x-ray crystallography, presumably because of a high degree of intrinsic disorder, which we confirmed using the IUPred prediction algorithm (29). We found that all known reviewed Gγ subunits, including the yeast Gγ subunit Ste18, exhibit intrinsic disorder throughout the first 8 to 15 residues of their N termini (Fig. 5*C*), which we visualized using a threaded structural model of the gamma subunit in the 1GP2 crystal structure (Fig. 5*D*). Thus, much like the disordered C-terminal tails of GPCRs (30), the intrinsically disordered N-terminal tails of Gγ subunits are phosphorylation hotspots in the canonical heterotrimeric G protein complex.

##### N-terminal Phosphorylation of the Yeast Gγ Subunit, Ste18, is Biologically Responsive and Essential for the Maintenance of Protein Steady State Level

**Fig. 6.**
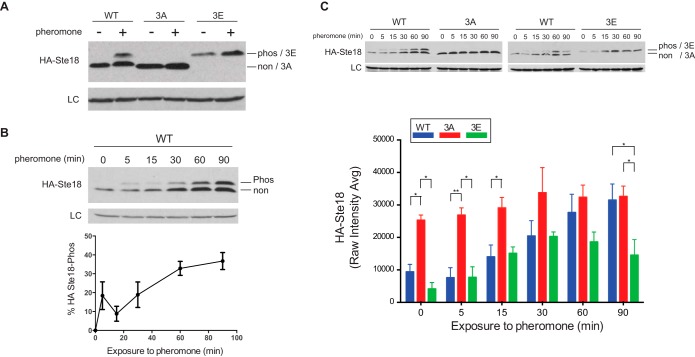
**The yeast Gγ subunit, Ste18, is phosphorylated in its N terminus in response to a GPCR stimulus and regulates protein abundance *in vivo*.** *A*, Immunoblot of HA-tagged Ste18 (WT), Ste18–3A (3A), and Ste18–3E (3E) in the absence and presence of α-factor mating pheromone. Cells were exposed to 3 μm pheromone for 60 min. *B*, Pheromone-induced phosphorylation kinetics of HA-Ste18 demonstrate an increase in the percentage of phosphorylation with time. *C*, The abundance of WT, 3A, and 3E phosphosite mutants was compared by immunoblotting at time intervals after pheromone stimulation. Error bars represent standard error of the mean for three independent experiments conducted on three different yeast colony isolates. Statistical significance was determined by 2-way ANOVA for the three independent experiments. G6PDH loading control (LC).

The budding yeast, *Saccharomyces cerevisiae*, is a longstanding model system for the study of G protein signaling and has been instrumental in the discovery of new regulators of G protein signaling including RGS proteins as well as PTM-based regulators (20, 21, 31–39). Signaling is activated when peptide pheromones (α-factor) bind the yeast GPCR (Ste2) at the plasma membrane, which activates a single canonical heterotrimeric G protein consisting of Gα (Gpa1), Gβ (Ste4) and Gγ (Ste18) subunits. Like other Gγ subunits, Ste18 harbors a disordered N-terminal tail (Ste18-Nt), phosphorylation of which has been observed by high throughput mass spectrometry but never tested for function experimentally (19). We tested the SAPH-ire prediction that Ste18-Nt phosphorylation would be biologically responsive and/or functional *in vivo*. First, we probed the electrophoretic mobility of wild-type Ste18 (HA-Ste18) by immunoblotting in the presence and absence of yeast mating pheromone (α-factor). In many cases, phosphorylation slows the electrophoretic mobility of proteins in comparison to their nonphosphorylated forms. The effect can be very pronounced for smaller proteins such as Gγ subunits. We found that HA-Ste18 exhibited a prominent mobility shift after pheromone stimulation (Fig. 6*A*). In contrast, the electrophoretic mobility of a phospho-null mutant (HA-Ste18–3A; see experimental procedures) was unaltered in the absence or presence of mating pheromone. Substitution of phospho-mimic mutations (HA-Ste18–3E) shifted the entire protein population upward in a manner similar to phosphorylation (Fig. 6*A*). We then measured the phosphorylation-dependent mobility shift as a function of time in the presence of pheromone. Ste18-Nt phosphorylation was rapidly activated within 5 min of pheromone stimulation, increasing proportionally with prolonged exposure to the stimulus (Fig. 6*B*). Thus, the N-terminal tail of the yeast Gγ subunit, Ste18, is phosphorylated in response to a biological stimulus.

Phosphorylation of yeast G proteins (Gα and Gβ) has been shown to regulate protein stability (21, 36, 40). Therefore, we determined if phosphorylation of Ste18-Nt was important for the stability of Ste18 by comparing the steady state levels of HA-Ste18 wild type (WT), phospho-null (3A) and phospho-mimic (3E) mutants in response to pheromone stimulation. We found that the abundance of wild-type Ste18 is low in the absence of pheromone but increases proportionally with exposure time to pheromone, resulting in an overall ∼threefold increase in protein abundance (Fig. 6*C*). Phospho-null mutations in Ste18-Nt resulted in significantly elevated (∼2.7-fold) protein levels under basal conditions and very little increase (∼1.3-fold) in response to pheromone (Fig. 6*C*). In contrast, phospho-mimic mutations exhibited a wild-type-like phenotype, with a low basal abundance and a pheromone-dependent increase in total protein level. Unlike wild type, the phospho-mimic mutant reached an abundance plateau after 30 min exposure to pheromone. Thus, Gγ-Nt phosphorylation is critical for dynamic maintenance of Ste18 steady state levels. We further conclude that Gγ phosphorylation hotspots predicted to have high function potential by SAPH-ire are valid for Ste18, a yeast-specific member of the Gγ protein family.

## DISCUSSION

PTMs intrinsically alter protein structure. The potential for a PTM to affect biological function is therefore directly related to its structural context. A systematic method that integrates structural context into the functional prioritization of PTM hotspots has been lacking. This is due in part to the ease of comparing sequence features by alignment and the difficulty of comparing protein features in three dimensions. Indeed, through this work we discovered numerous challenges that had to be overcome to integrate sequence, PTMs and protein structure. The most predominant challenges are: (1) the variability in entry format for the Protein Data Bank; and (2) the lack of a comprehensive database for known (*i.e.* experimentally validated) PTM function. Surmounting these relatively straightforward issues should be achievable by improving the content and systematic integration of biological databases. Structural Analysis of PTM Hotspots (SAPH-ire) approaches this goal and has revealed the first-ever quantitative structural topology of PTMs for any protein family.

We have validated SAPH-ire in two ways: first, by comparison to other ranking methods for the distinction between PTM hotspots with known *versus* unknown function; and second, by testing function potential predictions experimentally using the yeast model system for G protein signaling. As a result, we have discovered a new regulatory element for heterotrimeric G proteins – one of the most deeply studied protein systems in biology. Importantly, SAPH-ire is independent of structure type and thus, can be expanded to the analysis of any protein family given the availability of sufficient PTM and structural evidence (Fig. 3*A*). Consequently, SAPH-ire can be used to identify new regulatory elements in other eukaryotic protein systems. SAPH-ire can also be used to prioritize PTM experiments for a single protein family of interest, as we have done here. Based on this study, we extrapolate that the function of greater than 90% of all PTM hotspots remains unknown. Therefore, SAPH-ire can play a significant role in predicting which PTMs are most likely to regulate the function of proteins—one of the greatest barriers to understanding how PTMs evolve to regulate biological mechanism.

Unlike previously reported PTM prioritization methods, SAPH-ire is unique in multiple ways. First, SAPH-ire integrates PTM data for each alignment position independently as opposed to an alignment range (± 2 amino acids) (12). Nevertheless, SAPH-ire FP plots capture the same range-type of information because closely spaced clusters of PTMs will be members of the same or closely spaced peaks in a FP plot (Fig. 4*C*). Additionally, the sequence conservation, surface accessibility, and protein interface residence can overcome diminished PTM counts in the FP scoring regime. Indeed, we found that ∼20% of PTM hotspots with high a FP score and with known function contained only one or two experimental observations of the PTM (Fig. 3*C*). Second, SAPH-ire hotspots are allowed to contain multiple types of PTM (*e.g.* phosphorylation and ubiquitination), which may result from amino acid variability or PTM plasticity at the hotspot. At least for these eight protein families, we found that most hotspots (84.2%) contained only a single type of modification (see supplemental Fig. S1*A*). Nearly sixfold less frequent were hotspots that contain two different types of PTM (14.2%), and even fewer (1.6%) contain as many as three. Comparing those hotspots with two or three different types of PTM, we found that the majority (49%) could be explained as sites where a single residue type could be modified in multiple ways (*e.g.* acetylation, methylation or ubiquitination of lysine) (see supplemental Fig. S1*B*). Other frequently observed, multi-PTM hotspots could be explained by the known functional association between the PTMs found in the hotspot. Approximately 39% of all multi-PTM hotspots contained both phosphorylation and ubiquitination, representing the most common PTM-PTM association in our analysis. Functionally linked phosphorylation and ubiquitination sites have been shown to localize within five residues of one another and recycling of these sites within a short structural motif may result in alignment of phosphosites with ubiquitination sites (12). Last, we find that 12% of the hotspots in this study contained multiple PTMs for which there was no single explanation. Some of these were located within unstructured protein termini, for which sequence alignment is often difficult, whereas others (hotspots for S-nitrosylation and phosphorylation) remain unclear. However, by taking into account multiple types of PTM at each hotspot SAPH-ire can reveal structural elements that have been co-opted to serve as “regulatory handles” for a given protein family. We speculate that multi-PTM hotspots may also provide insight into the interchangeability of PTM types within a hotspot that might be harnessed to rewire protein networks.

SAPH-ire FP scoring relies on solvent accessible surface area values calculated from a three-dimensional protein structure. We found that many crystal structures in the PDB were inappropriate for FP scoring because of a range of disqualifying criteria such as the inclusion of chimeric proteins or grossly truncated proteins. Our choice of crystal structures used in this study were made before we applied the SAPH-ire method and were based on a few criteria intended to maximize accuracy, including: maximum resolved chain length, maximum structural resolution, and high literature citation of the structure. FP scores generated from other crystal structures (which included multiple different family members/uniprot entries) were largely invariant, with the average FP score of PTM hotspots with known function still exhibiting well above the median FP score (see supplemental File S2). However, we also observed that the percentage of PTM hotspots above the median FP score and with a known function dropped slightly from 94% when analyzing a single structure, to 90.2% when using average FP score. In fact, one PTM hotspot with known function in the Ras family was dramatically reduced in FP score when using a single structure *versus* the average of multiple structures. We found this resulted from a change in the orientation of the hotspot sidechain for some, but not most Ras-family structures. Future versions of SAPH-ire may exploit such variability to detect PTM hotspots located at conformationally variable regions that might be indicative of PTM-modulated conformational switches.

Further improvement of SAPH-ire may benefit by integrating additional PTM hotspot features. For example, PTM hotspot analysis is currently restricted to static protein structures, which lack information about local structural dynamics that exist for proteins found in their native environments. Therefore, integrating molecular dynamics of PTM hotspots may further improve the prediction accuracy of FP scoring in so much that the dynamics accurately represent structures in their native environments. Integrating phylogenetic analysis of PTM hotspots may also be useful. In this case, the breakdown of PTM hotspots across a phylogenetic map of the protein family may highlight certain hotspots that may have low sequence conservation within the whole family, but high conservation within a specific evolutionary branch of the family. Although this level of detailed PTM analysis is intriguing, we found that restriction to empirically verified PTMs severely limits the approach. Thus, either releasing this restriction (*i.e.* to include predicted sites of modification) or increasing the quantity of PTM data would be required for a meaningful result. In some families where there is a plethora of PTM data (*e.g.* rhodopsin-like GPCRs), a phylogenetic approach may be more useful. Beyond exploiting features such as these, SAPH-ire in its current form may be helpful in identifying the structural diversity of modifications with known function and how they differ between protein families.

We empirically evaluated PTM hotspots predicted to have high function potential by SAPH-ire. Specifically, we demonstrated that Gγ subunits contain a structurally conserved, intrinsically disordered N-terminal tail (Gγ-Nt) that is enriched with phosphorylation hotspots with high FP scores (Figs. 4 and 5). Using the yeast model system, we further showed that phosphorylation of this tail in the yeast Gγ subunit, Ste18, is responsive to pheromone GPCR activation and also essential for regulating protein steady state level (Fig. 6). Because this is the first description of a phospho-regulatory element for Gγ subunits, understanding the signaling role of Ste18 phosphorylation, specifically, will be useful for understanding the fundamental role of Gγ subunits, and their intrinsically disordered N-terminal tails, generally. Our data suggest that phosphorylation plays a role in the degradation of Ste18, because blocking phosphorylation of Ste18-Nt results in maximum levels of the protein, equivalent to the levels observed after 90-min pheromone stimulation; whereas mimicking phosphorylation results in wild-type protein levels (Fig. 6*C*). *STE18* gene expression does not appear to change with pheromone stimulation as determined by microarray analysis (41). Thus, Ste18 abundance in this case must be controlled predominantly at the PTM level. Furthermore, the pheromone-dependent regulation of factors (*e.g.* ubiquitin ligase) that control Ste18 abundance may also play an important role. Further understanding of how Ste18-Nt phosphorylation impacts G protein signal transduction and pathway activation will require precise control of Ste18 phosphorylation and protein abundance to elucidate the detailed mechanism of function.

This work describes the first discovery of a conserved regulatory element for heterotrimeric G proteins mediated through phosphorylation of Gγ subunit N-terminal tails. Consequently, the data reveal that PTMs play a larger role in regulating the signaling process than once thought. The G protein signaling mechanism is one of the most extensively studied to date and considerable evidence for the role of Gα subunits in GPCR-mediated signal transduction has been established. In contrast, a better understanding of the role of Gβ and Gγ subunits in signal transduction is still emerging. As an obligate heterodimer, the functional impact of Gβ and Gγ subunits has rarely been distinguished independently. Indeed, Gγ subunits are commonly thought to have limited functionality as membrane anchors for Gβ subunits. In this role, Gγ subunits require the prenylation of cysteine residues in their C-terminal tails (Function Potential = 22,870 and 55-fold over median-FP) and two alpha helices that stabilize the interaction with Gβ subunits (42–44). In the past decade, mammalian gene knockout models have linked specific Gγ subunits to a variety of distinct functions and disease states (45–52). In many cases, post-translational regulatory elements have been suggested, though never proven to participate in these critical roles of Gγ function. The intrinsically disordered N-terminal tails of Gγ subunits may also participate in these processes. Further work will be necessary to elucidate the precise role of Gγ-Nt phosphorylation in yeast, and also human systems from which the majority of PTM data originates.

## Supplementary Material

Supplemental Data

## Footnotes

^1^ The abbreviations used are:

PTM: post-translational modification
PDB: protein data bank
FP: function potential
MSA: multiple sequence alignment
GPCR: G protein coupled receptors.
