# Supplementary material for: Structural Analysis of PTM Hotspots (SAPH-ire) – A Quantitative Informatics Method Enabling the Discovery of Novel Regulatory Elements in Protein Families
Source: Mol Cell Proteomics. 2015 Jun 12;14(8):2285–97. doi: 10.1074/mcp.M115.051177 (PMC4528253; doi:10.1074/mcp.M115.051177)
Supplement: Supplemental Data [file supp_14_8_2285__index.html]

Structural Analysis of PTM Hotspots (SAPH-ire) – A Quantitative Informatics Method Enabling the Discovery of Novel Regulatory Elements in Protein Families — New Discovery of G-protein PTM-based Regulation Mechanism — Supplemental Data 

# Structural Analysis of PTM Hotspots (SAPH-ire) – A Quantitative Informatics Method Enabling the Discovery of Novel Regulatory Elements in Protein Families

## Supplemental Data

**Files in this Data Supplement:**

- Supplemental Tables and Figures - Supplemental Tables and Figures
- FP Heatmaps from multiple structures - Function Potential scores based on surface area data from multiple crystal structures for each family.
- PyMol Session Files - Eight PyMol session files (.pse) showing PTM hotspots on crystal structures (one for each family is shown).
- PTMs and FP Data
