# Supplementary material for: Structural Analysis of PTM Hotspots (SAPH-ire) – A Quantitative Informatics Method Enabling the Discovery of Novel Regulatory Elements in Protein Families
Source: Mol Cell Proteomics. 2015 Jun 12;14(8):2285–97. doi: 10.1074/mcp.M115.051177 (PMC4528253; doi:10.1074/mcp.M115.051177)
Supplement: Supplemental Data [file supp_M115.051177_Torres_Supplementary_Information_MCP.pdf]

Supplementary Information

Structural Analysis of PTM Hotspots (SAPH-ire) – A Quantitative Informatics Method Enabling the Discovery of Novel Regulatory Elements in Protein Families

Running Title: New Discovery of G-protein PTM-based Regulation Mechanism

Henry Dewhurst, Shilpa Choudhury, Matthew Torres*

SUPPLEMENTARY INFORMATION (This PDF File)

Supplementary Table 1. Table of PTM databases used in this study.

Supplementary Table 2. Table of PTMs analyzed by SAPH-ire in this study.

Supplementary Table 3. Table of PTM hotspots with known function and citations.

Supplementary Table 4. Table of oligonucleotides used for yeast strain creation.

Supplementary Table 5. Table of yeast strains used or created for this study.

Supplementary Figure 1. Analysis of multi-type PTM hotspots generated by SAPH-ire. (**a**) Number of hotspots comprised of 1, 2 or 3 different types of PTMs (percentage of total shown). (**b**) Heatmap of di-type PTM hotspots observed in this study (only PTMs found in multi-type PTM hotspots were analyzed for this figure).

ADDITIONAL SUPPLEMENTARY FILES (Files attached independently)

Supplementary File 1 (.xlsx). SAPH-ire data output with function potential scores and statistics for PTM hotspot ranking tests (*see Fig. 2*).

Supplementary File 2 (.xlsx). Function Potential scores based on surface area data from multiple crystal structures for each family.

Supplementary Files 3 – 10 (.zip). Eight PyMol session files (.pse) showing PTM hotspots on crystal structures (one for each family is shown).

Supplmental Table 1: Table of PTM Database Resources Used in this Study

**dbSNO:** Lee, T. Y., Chen, Y. J., Lu, C. T., Ching, W. C., Teng, Y. C., Huang, H. Da, & Chen, Y. J. (2012). dbSNO: A database of cysteine S-nitrosylation. *Bioinformatics*, *28*, 2293–2295.
**HPRD:** Prasad, T. S. K., Goel, R., Kandasamy, K., Keerthikumar, S., Kumar, S., Mathivanan, S., Telikicherla, D., Raju, R., Shafreen, B., Venugopal, A., Balakrishnan, L., Marimuthu, A., Banerjee, S., Somanathan, D. S., Sebastian, A., Rani, S., Ray, S., Kishore, C. J. H., Kanth, S., Ahmed, M., Kashyap, M., Mohmood, R., Ramachandra, Y. L., Krishna, V., Rahiman, A. B., Mohan, S., Ranganathan, P., Ramabadran, S., Chaerkady, R. and Pandey, A. Human Protein Reference Database - 2009 update. *Nucleic Acids Research*. 37, D767-D772.
**GGBase:** https://gygi.med.harvard.edu/ggbase/. 20 October 2014.
**MeMo:** Chen, H., Xue, Y., Huang, N., Yao, X., & Sun, Z. (2006). MeMo: A web tool for prediction of protein methylation modifications. *Nucleic Acids Research*, *34*. doi:10.1093/nar/gkl233
**OGlycBase:** Gupta, R., Birch, H., Rapacki, K., Brunak, S., & Hansen, J. E. (1999). O-GLYCBASE version 4.0: A revised database of O-glycosylated proteins. *Nucleic Acids Research*. doi:10.1093/nar/27.1.370
**Phospho.ELM:** Dinkel, H., Chica, C., Via, A., Gould, C. M., Jensen, L. J., Gibson, T. J., & Diella, F. (2011). Phospho.ELM: A database of phosphorylation sites-update 2011. *Nucleic Acids Research*, *39*.
**PhosphoGRID:** Sadowski, I., Breitkreutz, B.-J., Stark, C., Su, T.-C., Dahabieh, M., Raithatha, S., … Tyers, M. (2013). The PhosphoGRID Saccharomyces cerevisiae protein phosphorylation site database: version 2.0 update. *Database : The Journal of Biological Databases and Curation*, *2013*, bat026.
**PhosphoSitePlus:** Hornbeck, P. V., Kornhauser, J. M., Tkachev, S., Zhang, B., Skrzypek, E., Murray, B., … Sullivan, M. (2012). PhosphoSitePlus: A comprehensive resource for investigating the structure and function of experimentally determined post-translational modifications in man and mouse. *Nucleic Acids Research*, *40*.
**PupDB:** Tung, C.W. (2012). PupDB: a database of pupylated proteins. *BMC Bioinformatics*.
**Swiss-Prot:** Boeckmann, B., Bairoch, A., Apweiler, R., Blatter, M. C., Estreicher, A., Gasteiger, E., … Schneider, M. (2003). The SWISS-PROT protein knowledgebase and its supplement TrEMBL in 2003. *Nucleic Acids Research*.
**SysPTM:** Li, J., Jia, J., Li, H., Yu, J., Sun, H., He, Y., … Xie, L. (2014). SysPTM 2.0: an updated systematic resource for post-translational modification. *Database : The Journal of Biological Databases and Curation*, *2014*, bau025.
**UbiProt:** Chernorudskiy, A. L., Garcia, A., Eremin, E. V, Shorina, A. S., Kondratieva, E. V, & Gainullin, M. R. (2007). UbiProt: a database of ubiquitylated proteins. *BMC Bioinformatics*, *8*, 126.

Supplmental Table 2: Table of PTMs Analyzed by SAPH-ire in this Study

Name	Gα	Gβ	Gγ	α-Tub	β-Tub	Rho	Rab	Ras	Totals
Family	IPR001019	IPR016346	IPR001770	IPR002452	IPR002453	IPR003578	IPR003579	IPR020849	
Members	43	16	20	34	33	43	122	68	379
Total PTMs	169	46	66	389	305	139	391	223	1728
acetylation	1	6	8	45	6	6	24	7	103
ADP-ribosylation	0	1	0	0	0	1	0	3	5
blocked amino	0	0	1	0	0	0	0	0	1
caspase	0	0	0	0	0	0	1	0	1
deacetylation	0	0	0	1	0	0	0	0	1
deamidation	1	0	6	0	0	0	0	0	7
lipoprotein	0	0	0	0	0	0	0	1	1
methylation	0	0	0	10	3	6	5	12	36
myristoylation	10	0	0	0	0	0	1	0	11
Nucleotide-binding	0	0	0	0	0	6	4	0	10
nitration	0	0	0	2	0	0	0	0	2
palmitoylation	19	0	0	0	0	7	1	8	35
phosphorylation	77	24	34	223	216	51	248	143	1016
prenylation	0	0	10	0	0	10	38	20	78
proteolytic cleavage	0	0	1	1	0	2	1	0	5
S-nitrosylation	5	8	1	25	18	17	10	9	93
ubiquitylation	56	7	5	82	62	33	58	20	323

Supplmental Table 3: Table of PTMs with Known Function and Citations.

Family	Protein	Alignment Target	Alignment Target Residue	Function Potential Score	Pubmed References
IPR001019	G-alpha	P10824	2	21428	7536745, 1436039, 1936988, 8702760, 7822269
IPR001019	G-alpha	P10824	3	25324	7536745, 1436039, 1936988, 8702760, 7822269
IPR001019	G-alpha	P10824	16	724.64	7559455, 8429024, 9166747
IPR001019	G-alpha	P10824	29	1070.1	1378615, 7530445, 7559455, 8429024, 9166747
IPR001019	G-alpha	P10824	181	621.44	12399457
IPR001019	G-alpha	P10824	206	2605.68	8053895
IPR001019	G-alpha	P10824	314	959.36	12647293
IPR001019	G-alpha	P10824	351	974	12799383, 9188537
IPR016346	G-beta	P62871	129	1943.68	10734087
IPR016346	G-beta	P62871	204	494.4	20881007
IPR016346	G-beta	P62871	266	1759.2	12486123
IPR001770	G-gamma	P63212	68	21440.4	7822269, 10712512
IPR002452	alpha-tub	P09733	41	4432.4	reviewed in 20930140
IPR002452	alpha-tub	P09733	61	531.08	reviewed in 20930140
IPR002452	alpha-tub	P09733	113	7857.36	reviewed in 20930140
IPR002452	alpha-tub	P09733	164	18293.6	reviewed in 20930140
IPR002452	alpha-tub	P09733	165	2420.4	reviewed in 20930140
IPR002452	alpha-tub	P09733	312	5479.68	reviewed in 20930140
IPR002452	alpha-tub	P09733	327	16596.8	reviewed in 20930140
IPR002452	alpha-tub	P09733	371	6304	reviewed in 20930140
IPR002452	alpha-tub	P09733	395	11124.96	reviewed in 20930140
IPR002452	alpha-tub	P09733	402	13164.32	reviewed in 20930140
IPR002452	alpha-tub	P09733	447	2024.64	reviewed in 20930140
IPR002453	beta-tub	P02557	58	8711.2	reviewed in 20930140
IPR002453	beta-tub	P02557	172	336.33	reviewed in 20930140
IPR020849	Ras	P01112	32	1825.92	reviewed in 22189424
IPR020849	Ras	P01112	41	1180.8	reviewed in 22189424
IPR020849	Ras	P01112	117	733.44	reviewed in 22189424
IPR020849	Ras	P01112	118	428.24	reviewed in 22189424
IPR020849	Ras	P01112	128	494.04	reviewed in 22189424
IPR020849	Ras	P01112	147	4781.44	reviewed in 22189424
IPR020849	Ras	P01112	165	1185.76	reviewed in 22189424
IPR020849	Ras	P01112	181	3753.9	reviewed in 22189424
IPR020849	Ras	P01112	184	2502.6	reviewed in 22189424
IPR020849	Ras	P01112	186	50052	reviewed in 22189424
IPR003578	Rho	P61586	2	796.89	15775972
IPR003578	Rho	P61586	26	621.12	24872548
IPR003578	Rho	P61586	34	14875.28	23027962
IPR003578	Rho	P61586	66	4466.56	23027962, 22163037
IPR003578	Rho	P61586	73	169.76	10617634, 17088251, 16831194
IPR003578	Rho	P61586	135	3322.56	23871831
IPR003578	Rho	P61586	149	2851.84	16540523, 12437928, 12117911, 18093184
IPR003578	Rho	P61586	188	531.26	12736149, 12640036, 15890975, 21572420
IPR003578	Rho	P61586	189	1062.52	23622247
IPR003578	Rho	P61586	190	38250.72	22992742, 11523996, 15864282
IPR003579	Rab	P62491	75	5821.2	24872409
IPR003579	Rab	P62491	114	843.3	24872409, 10403367
IPR003579	Rab	P62491	145	917.82	24190883
IPR003579	Rab	P62491	177	2625.36	22188018
IPR003579	Rab	P62491	212	35004.8	12802062
IPR003579	Rab	P62491	213	19252.64	12802062

Supplmental Table 4: Table of Oligonucleotides Used for Yeast Strain Construction.

Oligo ID	Forward Primer	Reverse Primer
**ste18Δ-CORE**	CAATTTTAGGATAGTAGCAATCGCAAACGTTCTCAATAATTCTAAGAGAGCTCGTTTTCGACACTGG	GATATGAAAAAGCATGTATATTTTTTTTTTTTTTGGATTCTATTACTATCATCCTTACCATTAAGTTGATC
**QuikChange-pZM552-STE18-3A**	CAGCTGACCACCATGGCTGCTGTTCAAAACGCTCCACGCTTACAACAAC	GTTGTTGTAAGCGTGGAGCGTTTTGAACAGCAGCCATGGTGGTCAGCTG
**QuikChange-pZM552-STE18-3E**	CAGCTGACCACCATGGAAGAAGTTCAAAACGAACCACGCTTACAACAACC	GGTTGTTGTAAGCGTGGTTCGTTTTGAACTTCTTCCATGGTGGTCAGCTG
**STE18-3A-Popout**	GTCAATTTTAGGATAGTAGCAATCGCAAACGTTCTCAATAATTCTAAGAATGGCTGCTGTTCAAAACGC	ATGAAAAAGCATGTATATTTTTTTTTTTTTTGGATTCTATTACTATCATTACATAAGCGTACAACAAACAC
**STE18-3E-Popout**	GTCAATTTTAGGATAGTAGCAATCGCAAACGTTCTCAATAATTCTAAGAATGGAAGAAGTTCAAAACGAACCAC	ATGAAAAAGCATGTATATTTTTTTTTTTTTTGGATTCTATTACTATCATTACATAAGCGTACAACAAACAC
**CORE-STE18-WT**	GATAGTAGCAATCGCAAACGTTCTCAATAATTCTAAGAATGGAGCTCGTTTTCGACACTGG	CCTGAGGTTGTTGTAAGCGTGGAGAGTTTTGAACTGATGTTCCTTACCATTAAGTTGATC
**CORE-STE18-3A**	GATAGTAGCAATCGCAAACGTTCTCAATAATTCTAAGAATGGAGCTCGTTTTCGACACTGG	GTTCCTGAGGTTGTTGTAAGCGTGGAGCGTTTTGAACAGCAGCTCCTTACCATTAAGTTGATC
**CORE-STE18-3E**	GATAGTAGCAATCGCAAACGTTCTCAATAATTCTAAGAATGGAGCTCGTTTTCGACACTGG	GTTCCTGAGGTTGTTGTAAGCGTGGTTCGTTTTGAACTTCTTCTCCTTACCATTAAGTTGATC
**HA-STE18-WT**	GATAGTAGCAATCGCAAACGTTCTCAATAATTCTAAGAATGTATCCATACGACGTACCTGACTACGCT	CCTGAGGTTGTTGTAAGCGTGGAGAGTTTTGAACTGATGTAGCGTAGTCAGGTACGTCGTATGGATA
**HA-STE18-3A**	GATAGTAGCAATCGCAAACGTTCTCAATAATTCTAAGAATGTATCCATACGACGTACCTGACTACGCT	GTTCCTGAGGTTGTTGTAAGCGTGGAGCGTTTTGAACAGCAGCAGCGTAGTCAGGTACGTCGTATGGATA
**HA-STE18-3E**	GATAGTAGCAATCGCAAACGTTCTCAATAATTCTAAGAATGTATCCATACGACGTACCTGACTACGCT	GTTCCTGAGGTTGTTGTAAGCGTGGTTCGTTTTGAACTTCTTCAGCGTAGTCAGGTACGTCGTATGGATA

Supplmental Table 5: Table of Yeast Strains Used or Created for this Study.

Strain	Genotype	From
BY4741	*MAT**a** leu2Δ met15Δ his3Δ ura3Δ*	
ste18Δ::CORE	*BY4741 ste18::KanMX4-URA3*	This study
ste18-3A	*BY4741 ste18-3A*	This study
ste18-3E	*BY4741 ste18-3E*	This study

a

b

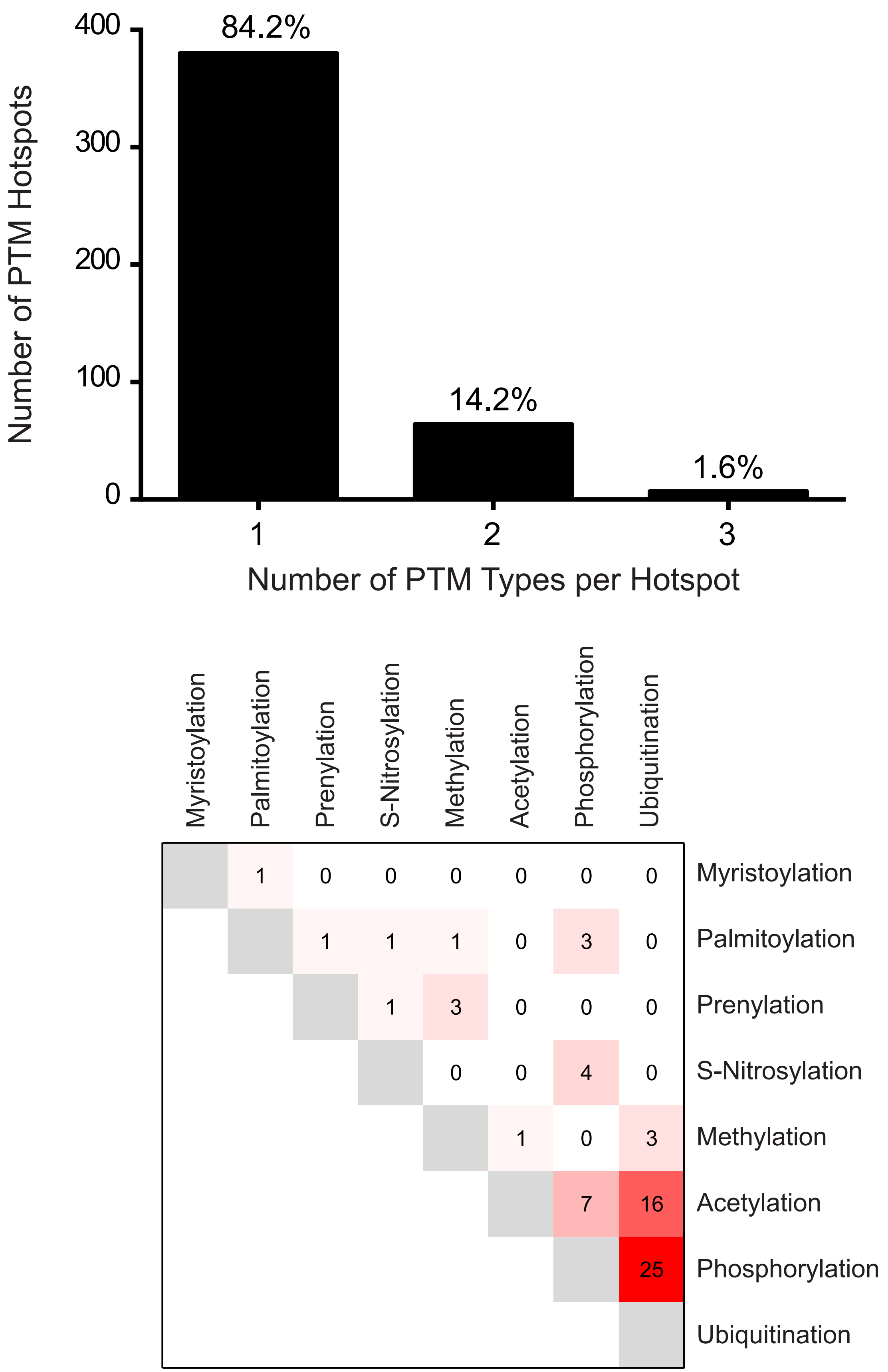

Supplementary Figure 1. Analysis of multi-type PTM hotspots generated by SAPH-ire. (a) Number of hotspots comprised of 1, 2 or 3 different types of PTMs (percentage of total shown). (b) Heatmap of di-type PTM hotspots observed in this study (only PTMs found in multi-type PTM hotspots were analyzed for this figure).
